# KLF10 Functions as an Independent Prognosis Factor for Gastric Cancer

**DOI:** 10.3390/medicina58060711

**Published:** 2022-05-26

**Authors:** Yueh-Min Lin, Kun-Tu Yeh, Chung-Min Yeh, Maw-Soan Soon, Li-Sung Hsu

**Affiliations:** 1 Department of Surgical Pathology, Changhua Christian Hospital, Changhua 500, Taiwan; 93668@cch.org.tw (Y.-M.L.); 10159@cch.org.tw (K.-T.Y.); 28935@cch.org.tw (C.-M.Y.); 2 School of Medicine, Chung Shan Medical University, Taichung 402, Taiwan; 3 Department of Post-Baccalaureate Medicine, College of Medicine, National Chung Hsing University, Taichung 402, Taiwan; 4 Department of Gastroenteology, Kuang Tien General Hospital, Taichung 433, Taiwan; 5 Department of Biology, Graduate Institute of Biotechnology, National Changhua University of Education, Changhua 500, Taiwan; 6 General Education Center, Chienkuo Technology University, Changhua 500, Taiwan; 7 Institute of Medicine, Chung Shan Medical University, Taichung 402, Taiwan; 8 Department of Medical Research, Chung Shan Medical University Hospital, Taichung 402, Taiwan

**Keywords:** KLF10, gastric cancer, prognosis

## Abstract

*Background and Objectives:* Krűppel-like factor 10 (KLF10) participates in the tumorigenesis of several human cancers by binding to the GC-rich region within the promoter regions of specific genes. KLF10 is downregulated in human cancers. However, the role of KLF10 in gastric cancer formation remains unclear. *Materials and Methods:* In this study, we performed immunohistochemical staining for KLF10 expression in 121 gastric cancer sections. *Results*: The loss of KLF10 expression was correlated with advanced stages and T status. Kaplan–Meier analysis revealed that patients with higher KLF10 levels had longer overall survival than those with lower KLF10 levels. Univariate analysis revealed that in patients with gastric cancer, advanced stages and low KLF10 levels were associated with survival. Multivariate analysis indicated that age, gender, advanced stages, and KLF10 expression were independent prognostic factors of the survival of patients with gastric cancer. After adjusting for age, gender, and stage, KLF10 expression was also found to be an independent prognostic factor in the survival of patients with gastric cancer. *Conclusion:* Our results collectively suggested that KLF10 may play a critical role in gastric cancer formation and is an independent prognosis factor of gastric cancer.

## 1. Introduction

Krűppel-like factors (KLFs) are highly conserved zinc-finger proteins that regulate cellular transcription machinery [1,2]. KLFs regulate a wide range of cellular functions, including cell proliferation, apoptosis, differentiation, and neoplastic transformation, by binding to GC-rich promoter regions [1,2].

Subramaniam et al. first identified KLF10, which was previously named as *tumor growth factor-beta (TGF-beta)-inducible early gene (TIEG1)*, from a human osteoblast cDNA library [3]. Recently, reports demonstrate that KLF10 play a critical role in human carcinogenesis [4,5,6]. In TGF-beta-resistant pancreatic cancer cells, the ectopic expression of KLF10 increases chemosensitivity to gemcitabine and induces apoptosis [7]. Hsu et al. demonstrated that the overexpression of KLF10 results in the reduction in Bax-inhibitor-1 expression and disrupts intracellular calcium homeostasis, which leads to the apoptosis of estrogen-dependent adenocarcinoma cells [8]. KLF10 recruits HDAC to suppress the expression of epithelial growth factor receptor (EGFR), and as a consequence, represses the EGF signals that are involved in the invasion and metastasis of breast cancer [9]. However, the association between the expression of KLF10 and the clinical outcomes of patients with cancer remains controversial. The cirFUT8, a circular RNA, enhances the expression of KLF10 and subsequently reduces the metastasis of bladder cancer [10]. The downregulation of KLF10 in pancreatic ductal adenocarcinoma stably expressing KRasG12D promotes invasion and metastasis via the activation of the SDF-1/CXCR4 and AP-1 pathways [11]. KLF10 is significantly diminished in multiple myeloma relative to in normal tissues [12]. KLF10 attenuates the nuclear translocation of beta-catenin, inhibits the expression of PTTG1, and then decreases the proliferation of multiple myeloma [12]. Expression of KLF10 was associated with a higher 5-year survival rate and was an independent factor for the overall survival rate [13]. Yang et al. demonstrated that increased KLF10 and then downregulation of EGFR enhanced the sensitivity of gemcitabine-resistant cholangiocarcinoma to photodynamic therapy [14]. In a randomized phase III trial, higher expression of KLF10 was correlated with better overall survival and recurrence-free survival [15]. Downregulation of KLF10 was found in multiple myeloma tissues compared to normal groups, whereas overexpression of KLF10 mitigated the proliferation of multiple myeloma cells by controlling PTTG1 expression [12]. By contrast, KLF10 is highly expressed in prostate cancers [16]. Ivanov et al. detected high KLF10 expression in renal cell carcinoma, colon, lung, and small intestine cancers [17].

Gastric cancer has a high prevalence and is one of the leading causes of cancer-related deaths in the Asian region [18]. In accordance with histopathological analysis, gastric cancer can be divided into three subtypes: intestinal, diffuse, and mixed [18]. Each subtype has a distinct genetic background. For example, the germline mutation of E-cadherin is found in the diffuse type, whereas *Helicobacter pylori* infection is associated with intestinal-type gastric cancer [19]. Our previous reports have shown that several KLF proteins are correlated with gastric tumorigenesis. KLF5 and KLF8 expression is associated with advanced tumor stages and poor prognosis [20,21]. Although KLF10 is associated with several cancers, its role in gastric cancer remains to be elucidated. In this study, we demonstrated that KLf10 expression is associated with clinical stages and overall survival rate. Our findings provided evidence that KLF10 may function as a tumor suppressor gene in gastric cancer.

## 2. Materials and Methods

### 2.1. Sample Collection

A total of 121 gastric cancer patients who underwent radical subtotal gastrectomy from 1999 to 2005 were enrolled in this study. The inclusion criteria were the cases performed by radically subtotal gastrectomy. The exclusion criteria were the cases not performed by radically subtotal gastrectomy and neoadjuvant chemotherapy. Chemotherapy such as adriamycin and 5-fluorouracil was applied to the status of nodal metastasis. The postoperation nutrition was oral nutritional intervention including dietary advice, oral nutritional supplements, or both. Monthly follow-up was recommended. These conditions showed no relevant differences between patients with high and low levels of KLF10 would be sufficient.

Formalin-fixed and paraffin-embedded gastric cancer specimens were obtained from patients who underwent surgical resection at the Department of Surgery, Changhua Christian Hospital, and tissue blocks were obtained from the Department of Surgical Pathology, Changhua Christian Hospital. Stages and grades were classified in accordance with the TNM and World Health Organization classification systems. All samples were arranged in tissue array blocks. Histopathological and clinical data were obtained from the cancer registry of Changhua Christian Hospital. Disease-free survival was measured as the time interval between surgical operation and either the date of death or the end of follow-up. This research was approved by the internal review board of Changhua Christian Hospital.

### 2.2. Tissue Microarrays

A tissue core section (2 mm in diameter) from cancer and tumor-adjacent normal part tissues in each paraffin block was longitudinally cut and arranged into new paraffin blocks by using a fine steel needle to generate tissue microarrays (TMAs). A 4 µm section was stained with hematoxylin and eosin to confirm the presence of the morphologically representative areas of the original cancers.

### 2.3. Analysis of KLF10 Expression via Immunohistochemistry (IHC)

IHC analysis was performed by using the streptavidin peroxidase protocol. Briefly, 4 µm sections of the paraffin-embedded cancer tissues and paired noncancerous tissue sections were deparaffinized. After blocking with 3% H_2_O_2_, the sections were rehydrated, and the antigen was retrieved by heating at 100 °C for 20 min in 10 mM citrate buffer (pH 6.0). After incubation with the anti-KLF10 antibody (SC-130408, Santa Cruz Biotechnology, Santa Cruz, CA, USA) for 20 min at room temperature, the slides were incubated with a horseradish peroxidase/Fab polymer conjugate for an additional 30 min, then washed thoroughly three times with PBS. Color was developed by using 3,3′-diamino-benzidine tetrahydrochloride as a chromogen, and the samples were counterstained with hematoxylin. Appropriate positive and negative controls were also included in IHC staining. After staining, the slides were scored by two pathologists. The paraffin-embedded and fresh frozen sections of the normal colonic epithelium of a homogeneous immunophenotype for the studied antigens were included as the positive controls. Negative controls had the primary antibody omitted and replaced with PBS. The intensity of the cytoplasmic and nuclear staining of the KLF10 protein was scored semiquantitatively in accordance with the percentage of positive cells in individual lesions. Here, 0–10% KLF10 protein staining was defined as negative (−), and greater than 10% KLF10 protein staining was defined as positive (+) as previously described [22]. The staining intensity of the cytoplasm was scored from 0 to 4 as previously reported. Intensity was classified as either weak (<2) or strong (≥2) as previously described [23].

### 2.4. Statistical Analysis

The relationship between the KLF10 expression level and different clinicopathological parameters was determined through χ^2^ and Fisher exact tests. The overall survival rate was analyzed via Kaplan–Meier plot and log-rank test. Multivariate analysis based on a Cox proportional hazard regression model was used to evaluate the prognostic significance of the clinical variables. All statistical analyses were performed by using SPSS statistical software (version 15.0; SPSS Inc., Chicago, IL, USA). *p* < 0.05 was considered to be statistically significant.

## 3. Results

### 3.1. Association of KLF10 Expression with the Clinical Parameters of Patients with Gastric Cancer

**Figure 1:**
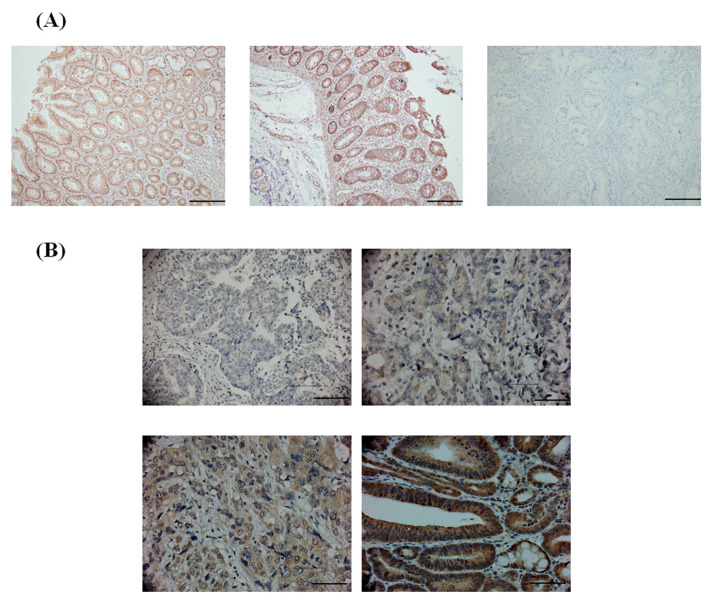
The expression patterns of KLF10 in gastric cancer. (**A**) Representative Immunohistochemistry (IHC) images of immunohistochemical staining of KLF10 gastric tissues. Left panel: IHC staining of normal gastric tissue. Middle panel: positive IHC staining region in normal gastric tissues. Right: IHC staining without KLF10 antibody in gastric tissues as negative control. All images magnification ×100. Scale bar: 5 µM. (**B**) Immunohistochemical staining of KLF10 in gastric cancer. KLF10 was positively detected in the nuclear and cytoplasmic regions. Representative IHC intensity of images of negative (upper panel, left), 1+ (upper panel, right), 2+ (lower panel, left) and 3+ (lower panel, right) were shown. All images magnification ×400. Scale bar: 20 µM.

Previous reports have shown that KLF10 may play a critical role in several human cancers [3]. We performed IHC staining by using gastric cancer TMAs to evaluate the relationship between the expression level of KLF10 and the clinical parameters of gastric cancer. The expression level of KLF10 was subdivided into two categories, namely, low and high, on the basis of IHC staining intensity as described in the Section 2 (Figure 1).

Significantly higher KLF10 expression was found in intestinal and mixed types than in diffused gastric cancer types (*p* < 0.001). KLF10 expression had an inverse relationship with tumor stages (*p* = 0.005) as shown in Table 1.

### 3.2. KLF10 Expression Was Correlated with the Survival of Patients with Gastric Cancer

**Figure 2:**
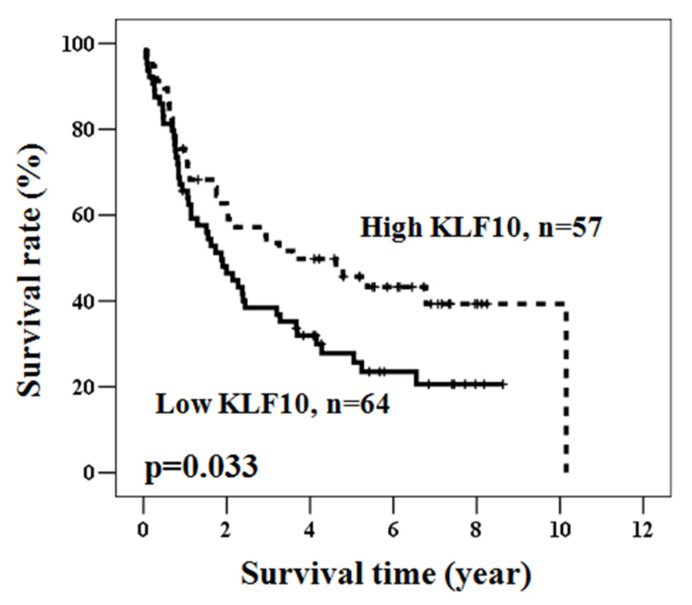
The prognostic significance of KLF10 in gastric cancer. Kaplan–Meier survival curve for the 121 gastric cancer patients with KLF10 protein expression. Patients with high KLF10 expression had a significantly better overall survival rate compared to patients with low KLF10 expression, as defined by a log-rank test (*p* = 0.033).

Next, the overall survival rate was analyzed through the Kaplan–Meier method to address whether KLF10 expression level was correlated with patient survival. As shown in Figure 2, overall survival was significantly longer in patients with higher KLF10 expression than in those with low KLF10 expression.

### 3.3. KLF10 Was an Independent Prognosis Factor of Gastric Cancer

We performed univariate Cox regression analysis to investigate the relationship between possible prognostic factors and patient survival. Advanced stages (hazard ratio = 3.723, 95% confidence interval = 1.615–8.583, *p* = 0.002) and low KLF10 expression (hazard ratio = 1.623, 95% confidence interval = 1.036–2.544, *p* = 0.035) were found to be prognostic factors (Table 2). Multivariate Cox regression analysis revealed that age, advanced stages, and low KLF10 expression were independent prognostic factors (Table 3). After adjusting for gender, age, and stage, a high KLF10 expression level was found to be significantly associated with a good overall survival rate of patients with advanced ages (≥71), both genders (female and male), intestinal-type gastric cancer, early stage (I + II), *t* value, and *n* value (Table 4).

## 4. Discussion

Gastric cancer is a high-mortality cancer in the Asian region [18]. KLF family proteins play an important role in several biological functions [5]. KLF10 may also be associated with several diseases [3]. In this study, IHC staining demonstrated that the loss of KLF10 was associated with advanced tumor stages and short survival. Univariate and multivariate analyses also revealed that KLF10 could be a prognostic factor of gastric cancer. Moreover, in the gastric cancer line, KLF10 overexpression altered the Bcl-2/Bax ratio, caused caspase-3 activation, and finally, caused apoptosis.

Decreased KLF10 expression has been found in several human cancers. Gene expression profiling analysis indicated that KLF10 expression is low in metastatic brain tumors [24]. Chang et al. demonstrated that KLF10 expression is downregulated by hypermethylation in pancreatic cancer [25]. The loss of KLF10 expression is correlated with pancreatic cancer stages. Multiple logistic regression analysis also revealed that KLF10 is an independent prognostic factor of prognosis-free survival and overall survival in pancreatic cancer [25]. In mice, KLF10 knockout facilitates skin tumorigenesis and papilloma formation in response to DMBA/TPA treatment [26]. Very recently, Yeh et al. demonstrated that KLF10 is an independent prognosis factor for oral squamous cell carcinoma [13]. In line with these observations, our findings indicated that KLF10 was low in gastric cancers with advanced stages. In addition, KLF10 was an independent factor in the overall survival of patients with gastric cancers. Heo et al. reported lower tumor incidence and proliferation of cell nuclear antigen in the liver of KLF10 null mice exposed to *N*-nitrosodiethylamine than in wild-type mice [27]. Our findings collectively suggested that KLF10 may be a tumor suppressor in gastric cancer.

Several proteins have been shown to be differentially expressed in the diffuse and intestinal types of gastric cancers. B cell translocation gene 1 is more likely expressed in the intestinal type than in the diffuse type of gastric cancer [28,29]. The mRNA of mitochondrial cytochrome c oxidase I and NADH dehydrogenase 4 is predominantly expressed in diffuse-type gastric cancer [30]. The loss of the expression of the breast cancer type 1 susceptibility protein is associated with the diffuse type of gastric cancer, whereas the loss of mediator of DNA damage checkpoint protein 1 is correlated with the intestinal type of gastric cancer [31]. Moreover, reports from our laboratory indicated that distinct KLF expression patterns are found in different types of gastric cancer. High nuclear KLF8 expression is observed in the diffuse type of gastric cancer [20]. In this study, KLF10 expression was significantly higher in intestinal-type gastric cancer than in other types. After adjusting for gender, age, and stage, KLF10 expression was found to be an independent prognostic factor of intestinal gastric cancer. Tanabe et al. found extensive epithelial-to-mesenchymal transition (EMT) in the diffuse type, but not the intestinal type, of gastric cancer [32]. This phenomenon results in the poor prognosis of diffuse-type gastric cancer [32]. By contrast, silencing KLF10 leads to the suppression of EMT protein expression [33]. The exact role of KLF10 in the development of intestinal-type gastric cancer remains to be investigated. Our results collectively revealed that KLF10 may be involved in the carcinogenesis of gastric cancer, especially intestinal-type gastric cancer. Since the pathogenesis of gastric cancer involves several steps and several factors/co-factors, future studies should focalize on the relationship between KLF10 expression and some of these factors, for example, *Helicobacter pylori* infection.

## 5. Conclusions

In conclusion, our findings provided the first piece of evidence supporting the role of KLF10 in gastric carcinogenesis. KLF10 expression was inversely correlated with tumor stages. Furthermore, KLF10 expression was low in the diffuse type of gastric cancer. KLF10 was also an independent prognostic factor of gastric cancer, especially the intestinal type of gastric cancer.

## Figures and Tables

**Table 1:** Relationships of KFL10 expression with clinical parameters in 121 gastric cancer patients.

Parameters	Case Number	KFL10 Expression		*p*-Value
		Low (%)	High (%)	
Age (year)		69.4 ± 12.0	72.4 ± 13.4	0.194
Gender				
Female	35	20 (57.1)	15 (42.9)	0.550
Male	86	44 (51.2)	42 (48.8)	
Type				
Diffuse	23	21 (91.3)	2 (8.7)	<0.001
Intestinal	70	32 (45.7)	38 (54.3)	
Mix	28	11 (39.3)	17 (60.7)	
Stage				
I	19	13 (68.4)	6 (31.6)	0.005
II	31	10 (32.3)	21 (67.7)	
III	49	24 (49.0)	25 (51.0)	
IV	22	17 (77.3)	5 (22.7)	
*t* value				
1	14	8 (57.1)	6 (42.9)	0.055
2	16	5 (31.3)	11 (68.8)	
3	76	39 (51.3)	37 (48.7)	
4	15	12 (80.0)	3 (20.0)	
*n* value				
0	36	21 (58.3)	15 (41.7)	0.810
1	29	14 (48.3)	15 (51.7)	
2	25	12 (48.0)	13 (52.0)	
3	31	17 (54.8)	14 (45.2)	
*m* value				
0	116	61 (52.6)	55 (47.4)	0.745
1	5	3 (60.0)	2 (40.0)	
Grade				
Well	8	5 (62.5)	3 (37.5)	0.723
Moderate	45	22 (48.9)	23 (51.1)	
Poor	68	37 (54.4)	31 (45.6)	

**Table 2:** Univariate analysis of the influence of various parameters on overall survival in 121 gastric cancer patients.

Parameter	Category	Overall Survival			
		5-Year Survival (%)	Hard ratio (HR)	95% CI	*p*-Value
Age (year)	≥71/<71	31.7/41.2	1.433	0.919–2.233	0.112
Gender	Male/female	31.1/49.3	1.613	0.971–2.681	0.065
Stage	II + III + IV/I	29.3/72.9	3.723	1.615–8.583	0.002
KFL10 expression	Low/high	27.8/45.7	1.623	1.036–2.544	0.035

**Table 3:** Multivariate analysis of the influence of various parameters on overall survival in 121 gastric cancer patients.

Parameter	Category	Overall Survival			
		5-Year Survival (%)	HR	95% CI	*p*-Value
Age (year)	≥71/<71	31.7/41.2	2.314	1.443–3.712	<0.001
Gender	Male/female	31.1/49.3	1.561	0.935–2.605	0.088
Stage	II + III +I V/I	29.3/72.9	5.344	2.266–12.599	<0.001
KFL10 expression	Low/high	27.8/45.7	2.344	1.460–3.762	<0.001

**Table 4:** Multivariate analysis of the influence of KFL10 expression according to clinical parameters on overall survival in gastric cancer patients.

Parameter	Overall Survival ^1^			
	5-Year Survival (%)	HR	95% CI	*p*-Value
All cases	27.8/45.7	2.344	1.460–3.762	<0.001
Age (year)				
<71	32.5/55.7	2.117	0.984–4.556	0.055
≥71	21.3/39.3	2.637	1.436–4.844	0.002
Gender				
Female	41.7/59.3	3.001	1.129–7.977	0.028
Male	22.7/40.7	2.085	1.218–3.570	0.007
Type				
Diffuse	19.0/0.0	1.373	0.286–6.596	0.692
Intestinal	36.9/57.1	2.565	1.326–4.960	0.005
Mix	16.4/23.2	2.199	0.700–6.906	0.177
Stage				
I + II	45.2/66.2	5.138	2.064–12.793	<0.001
III + IV	18.8/25.9	1.467	0.815–2.642	0.201
*t* value				
1 + 2	59.3/70.6	22.757	3.377–153.357	0.001
3 + 4	19.8/34.6	1.762	1.051–2.953	0.032
*n* value				
0	44.4/73.3	4.676	1.447–15.111	0.010
1 + 2 + 3	20.3/35.5	1.898	1.117–3.227	0.018

^1^ Adjusted for age, gender, and stage.
